# Determinants of the Downward Trend in Coronary Artery Bypass Graft Surgery Among Patients With Multivessel Disease and Class-I Indication for Surgery

**DOI:** 10.7759/cureus.14098

**Published:** 2021-03-25

**Authors:** Jabar Ali, Fahad R Khan, Safi Khattak, Hidayat Ullah, Rizwan Ullah, Gul Lakhta

**Affiliations:** 1 Cardiology/Interventional Cardiology, Lady Reading Hospital, Peshawar, PAK; 2 Cardiology, Lady Reading Hospital, Peshawar, PAK; 3 Interventional Cardiology, National Institute of Cardiovascular Diseases, Karachi, PAK; 4 Gynecology and Obstetrics, Lady Reading Hospital, Peshawar, PAK

**Keywords:** trend, coronary artery bypass graft surgery, determinants, disease determinants, class-i indication, cabg surgery, multi-vessel disease, percutaneous intervention, downtrend, socio-economic factors

## Abstract

Introduction

Coronary artery bypass graft (CABG) is the most effective coronary revascularization procedure, and it has been endorsed by many trials and studies over the years. However, due to CABG's immediate adverse effects, patients tend to prefer percutaneous coronary intervention (PCI) for coronary revascularization over it. This article focuses on the recent downtrend in CABG procedures for revascularization among patients for whom it is indicated. This study’s main objective was to identify the factors responsible for the downtrend in patients undergoing CABG despite a clear indication for it in those with multivessel diseases.

Methods

This study was conducted at the Lady Reading Hospital, Peshawar, Pakistan, from August 1, 2020, to January 1, 2021. A total of 340 patients with a class-I indication (presence of conditions regarding which there is evidence and/or general agreement that a given procedure or treatment is beneficial, useful, and effective) for CABG were enrolled in the study. Data related to all the variables were collected from patients and hospital records through an adequately designed proforma. For analysis, we applied the chi-square test to elaborate on the data for information and point biserial correlation to rule out the effect of age and weight on CABG’s downward trend.

Results

The mean age of the patients was 58.77 ±* *9.54 years; 65.88% were male, and 34.12% were female. Only 17.65% of the patients underwent CABG; 71.47% opted for medical treatment, and 9.41% underwent PCI. Out of the 280 patients who did not undergo CABG, 26.76% had financial issues; 23.82% were high-risk patients and hence refused surgeries by the surgeons; 20.59% of patients were not willing to undergo surgery; 7.94% were on the waiting list, and 3.24% had deranged renal function tests (RFTs).

Conclusions

A limited number of patients underwent revascularization therapy even though they had clear indications for CABG. The high-risk status of patients, patients' unwillingness, and the cost of the procedure were the primary reasons behind the downtrend in CABG procedures among patients with a clear indication for the same.

## Introduction

Coronary artery bypass graft (CABG) is a major surgical procedure that involves bypassing diseased coronary arteries in patients with coronary artery stenosis. The aim is to restore blood flow to the ischemic myocardium, improve myocardial function, and alleviate angina symptoms. With nearly 400,000 CABG surgeries performed each year, it is the most commonly conducted major surgical operation. Nevertheless, there has been a recent downward trend in the number of CABGs performed due to the availability of alternative options such as medical therapy and percutaneous coronary intervention (PCI) [1].

However, even with the widespread indication for PCI and the increasingly high-risk patient profile [European System for Cardiac Operative Risk Evaluation (EuroSCORE) was used to measure operative risk; a score of more than 5 was considered high risk], CABG still remains the mainstay of treatment for multivessel diseases, with exceptional long-term results and low complication rates. CABG continues to be the benchmark for revascularization in patients with multivessel coronary artery disease (CAD) [2]. In patients with multivessel CAD, CABG’s advantage over medical therapy was identified by three randomized trials conducted more than three decades ago [3-5]. Even though CABG has emerged over the past several years as a promising and versatile procedure and remains the mainstay of treatment for multivessel diseases, it is not commonly practiced throughout the world in general and Pakistan in particular. A heart team strategy is vital for every patient with complex CAD to make the right decision related to the treatment [6]. Currently, there is scarce data and information on this subject in the literature. Annual PCI rates have plummeted from 366 to 180 per 100,000 US adults, and the number of CABGs performed has dropped significantly from 159 to 82 per 100,000 US adults [7-8].

A study by Tu et al. has shown a significant difference in the PCI-to-CABG utilization ratio among individual hospitals. The choice of a particular procedure was found to be determined by the physician's preference [9]. The factors responsible for the increase in patients choosing PCI and medical therapy over CABG are not widely reported and extensively explored in the literature. Because of the increasing prevalence of CAD and the need for revascularization, it is critical to understand the causal factors behind CABG surgery’s downtrend and it warrants further research. In light of these facts, our objective was to identify the factors responsible for the downtrend in CABG procedures. The significance of this research lies in the fact that it will hopefully enable health departments to identify and rectify the issues responsible for this decline.

## Materials and methods

This retrospective study was conducted in the Department of Cardiology, Lady Reading Hospital, Peshawar. Data were collected from the hospital's catheterization laboratory in Peshawar from August 1, 2020, till January 1, 2020. After obtaining approval from the hospital's Ethical Committee and informed consent from patients, a cross-sectional study was carried out involving all patients diagnosed with the multivessel disease with a class-I indication (presence of conditions indicating that treatment is useful and beneficial and outweighs the risk and should be done) for CABG. Among all coronary angiographies performed during these five months, 340 patients had a recommendation for CABG. The procedure was indicated for all patients who had angiographic findings of triple vessel disease (TVD), left main stem (LMS) stenosis, double vessel disease (DVD) along with diabetes, and left main equivalent disease [i.e., 70% or greater stenosis of the left anterior descending (LAD) and proximal left circumflex artery (LCX)], mostly if the left ventricular function was compromised. An adequately designed proforma was used for the data collection. For data storage and analysis, SPSS Statistics version 25 (IBM, Armonk, NY) was used. Continuous variables like age and weight were presented as means and standard deviations (SD). Categorical variables such as gender, echocardiographic findings, CABG indications, and the determinants behind the refusal of CABG were expressed in frequency and percentages. The Shapiro-Wilk test was performed to determine whether the distributions of age and weight were significantly different from a normal distribution which showed that the data was not normally distributed. Therefore, a two-tailed Mann-Whitney U test was conducted to examine whether there were significant differences in age and weight between those who underwent CABG and those who did not. The Fisher’s exact test was conducted to determine a statistically significant relationship between CABG and the determinants behind the refusal of CABG.

## Results

The mean age of patients enrolled in the study was 58.77 ± 9.54 years. Of the total 340 patients, 224 (65.88%) were male, and 116 (34.12%) were female. On echocardiography, 137 (40.29%) had moderately impaired (31-40%) left ventricle ejection fraction (LVEF), (20.29%) had an ejection fraction (EF) on the borderline (41-49%), 74 (21.76%) patients had severely impaired EF (less than 30%) and 60 (17.65%) patients had a normal EF. Baseline characteristics of the subjects are presented in Table 1.

**Table 1:** Baseline characteristics of the study subjects SD: standard deviation

Variables	Frequency	Mean ± SD	Percent
Age, years		58.77 ± 9.54	
Weight, kg		82.73 ± 16.39	
Gender			
Male	224		65.88
Female	116		34.12
Ejection fraction (EF)			
Severely impaired EF (less than 30%)	74		21.76
Moderately impaired EF (31-40%)	137		40.29
Borderline EF (41-49%)	69		20.29
Normal EF (50% and above)	60		17.65

Only 60 (17.65%) patients underwent CABG. Among the remaining 280 (82.35%) patients, 214 (62.94%) opted for medical therapy, while 66 (19.41%) patients underwent PCI, as shown in Table 2.

**Table 2:** Treatment options chosen by patients

Coronary artery bypass graft (CABG)	Frequency	Percent
Yes	60	17.65
No	280	82.35
Medications		
Yes	214	62.94
No	97	28.53
Percutaneous coronary intervention (PCI)		
Yes	66	19.41
No	274	80.59

**Figure 1:**
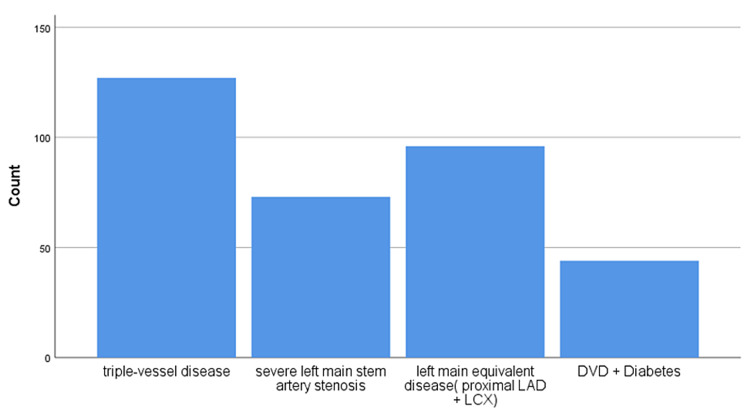
Bar chart showing indications for CABG CABG: coronary artery bypass graft; LAD: left anterior descending artery; LCX: left circumflex artery; DVD: double vessel disease

The main indications for CABG in our sample were TVD (127 patients, 37.35%), severe LMS artery stenosis (73 patients, 21.47%), left main equivalent disease (proximal LAD + LCX; 96 patients, 28.24%), and DVD with diabetes (44 patients, 12.94%), as shown in Table 3 and Figure 1.

**Table 3:** Indications for CABG CABG: coronary artery bypass graft; TVD: triple vessel disease; LMS: left main stem; LAD: left anterior descending artery; LCX: left circumflex artery; DVD: double vessel disease; DM: diabetes mellitus

Indications	Frequency	Percent
TVD	127	37.35
Severe LMS disease	73	21.47
Left main equivalent disease (proximal LAD + LCX)	96	28.24
DVD + DM	44	12.94

**Figure 2:**
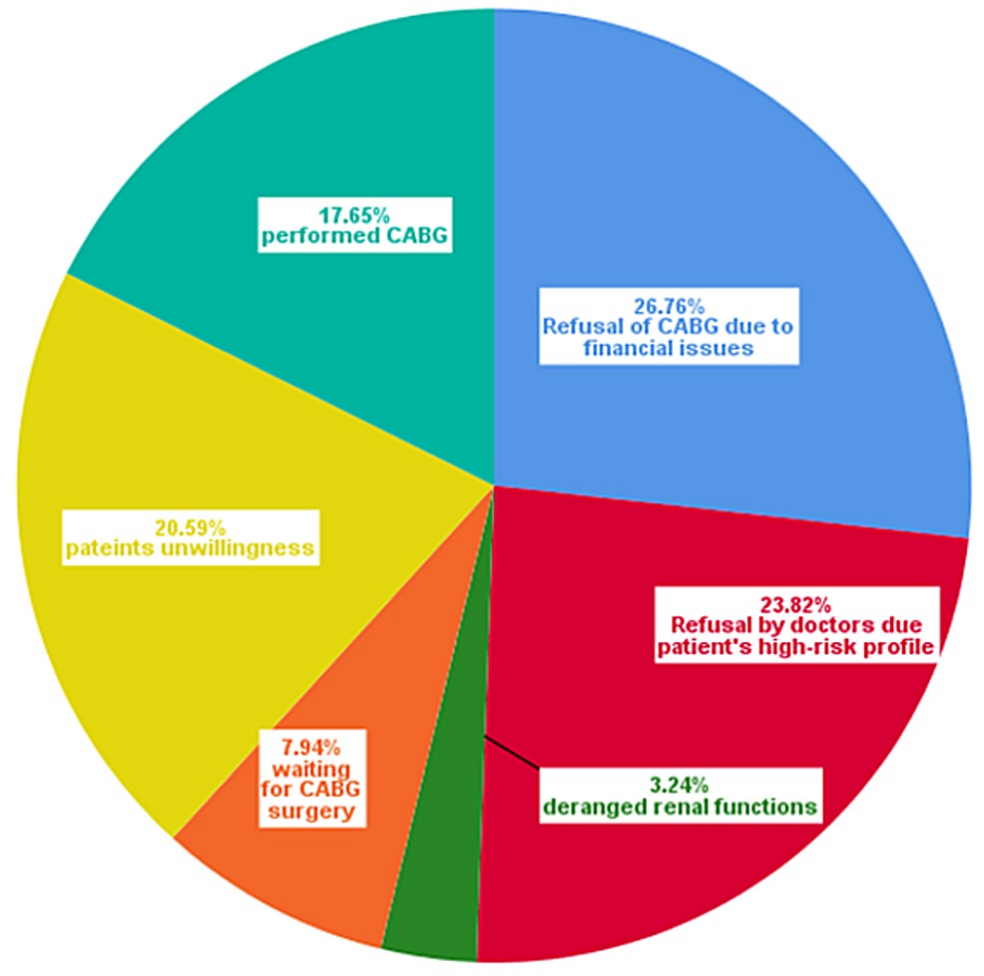
Determinants behind the refusal of CABG CABG: coronary artery bypass graft

Among the 280 patients without CABG, 91 (26.76%) could not afford CABG due to financial issues, 81 (23.82%) were deemed high-risk [European System for Cardiac Operative Risk Evaluation (EuroSCORE) of >5] due to which the doctor refused surgery, 70 (20.59%) patients were not willing to undergo open-heart surgery, 27 (7.94%) were on the waiting list for CABG, and 11 (3.24%) patients had deranged renal function tests (RFTs), as shown in the pie chart below (Figure 2).

The two-tailed Mann-Whitney U test results were not significant for both age and weight, suggesting no significant differences in age and weight between those who underwent CABG and those who did not. The results of the Mann-Whitney U tests are shown in Table 4.

**Table 4:** Two-tailed Mann-Whitney U test for age and weight by CABG CABG: coronary artery bypass graft

Variable	Mean rank		U	Z	P
	Yes	No			
Age	170.72	170.45	8413.5	-0.02	0.984
Weight	180.65	168.32	9009	-0.89	0.374

Fisher’s exact test results were significant based on an alpha value of 0.05, p: <.001, suggesting a statistically significant relationship between CABG and the determinants behind the refusal of CABG, as shown in Table 5.

**Table 5:** Relationship between CABG and determinants behind the refusal of CABG CABG: coronary artery bypass graft

Determinants behind the refusal of CABG	CABG		P	
	Yes	No		
Refusal of CABG due to financial issues	16.06%	74.94%		<0.001
Refusal by doctors due to the high-risk profile of patients	14.29%	66.71%		
Unwillingness among patients	12.35%	57.65%		
Waiting for CABG surgery	4.76%	22.24%		
Deranged renal functions	1.94%	9.06%		

## Discussion

Our study demonstrated that the trend of not opting for CABG among patients with a class-I indication can be attributed to many factors. In those patients who did not undergo CABG, two-thirds opted to continue medical treatment, and only 66 patients underwent PCI rather than CABG for symptomatic relief. The most common indication for CABG in our sample was TVD (127 patients, 37%) followed by left main equivalent disease (proximal LAD + LCX; 96 patients, 28.2%).

To our knowledge, this is the first study carried out to determine the factors behind the low rate of CABG in patients with clinical indications for the procedure. These findings are inconsistent with the analysis results from the Acute Coronary Treatment and Intervention Outcomes Network Registry in 2012, where most patients underwent either CABG or PCI (40% each), and only 20% did not undergo revascularization [10]. In contrast, in our study, 17.65% underwent CABG, 19.41% underwent PCI, and the remaining 62.94% did not undergo revascularization. As a result, the odds of CAD being treated with CABG vary significantly among the targeted populations. Besides, recent data analyzed over the last two decades have shown a downward trend in revascularization procedures, with CABG being more successful than PCI [11-13].

The importance of CABG cannot be denied despite the emergence of alternative technologies and minimally invasive procedures in medicine. In a Bypass Angioplasty Revascularization Investigation-2 Diabetes (BARI-2D) study, 2,368 diabetic patients with stable CAD were randomized to either PCI or CABG therapy. The study showed a decline in significant adverse cardiovascular events at five years in the CABG group [14]. In comparison, our study comprised both diabetics and non-diabetics. In the BARI-2D study, the revascularization group comprised 88.3% of patients, and the medical-therapy group comprised 87.8% of patients. In contrast, in our study, the revascularization group (CABG + PCI) had 37.06% of patients, and the medical group had 62.94% of patients. Even though we did not follow up on our patients, given the myriad advantages of CABG over PCI, our study laid stress on resolving the various factors responsible for the downward trend in CABG procedures.

In a study by Javed et al. involving 1,680 patients with multivessel CADs, randomized to the CABG surgery group and PCI group, the authors concluded that there were more adverse cardiovascular events in patients who underwent PCI compared to those who underwent CABG. Though this study preferred CABG surgery for diabetic patients, it does point toward CABG’s effectiveness [7]. Similarly, in another randomized trial conducted by Head et al. on 1,095 patients with TVD, CABG was determined to be the superior option, resulting in substantially lower major adverse cardiac events than PCI [8]. These studies have highlighted the importance of CABG surgery and its superiority over PCI; even then, only 17.65% of patients in our study underwent CABG surgery, while 19.41% of patients chose PCI over CABG. The remaining 62.94% opted for medical therapy. Our study has shed light on the various causal factors behind this downtrend in CABG surgery. Financial issues, the unwillingness among patients, and surgeons' refusal due to patients’ high-risk profile were the main factors responsible for CABG surgery’s downward trend.

People generally tend to opt for less invasive treatment, such as PCI, over more invasive methods, such as CABG, regardless of the long-term consequences. A hypothetical choice was given to the patients in a study to choose between PCI and CABG. They chose PCI over CABG, although they were advised beforehand that the mortality rate would be twice in the PCI scenario compared to CABG; this clearly indicates the overwhelming preference people show toward less invasive medical procedures [15]. In our study, 20.59% of patients did not undergo CABG surgery because of their unwillingness.

In another study conducted in Canada from 1994 to 2005, the authors focused on changing coronary revascularization strategies from CABG to PCI. They concluded that significant inter-regional and inter-hospital variations exist regarding this aspect, the causes of which remain uncertain [16]. Our study strove to fulfill this gap and focused on revealing the reasons behind the downtrend of CABG in Pakistan. Financial issues (26.76%) came out on top, followed by the refusal by surgeons due to the high-risk patient profiles (EuroSCORE of >5; 23.82%), the unwillingness among patients (20.59%), the excessive waiting time for CABG surgery (7.94%), and deranged RFTs (3.24%).

The change in trend indicating preference of medical therapy and PCI over CABG is attributed to improved medical therapy, considerable differences in the characteristics, the risk profile of patients, low income, the clinical presentation of patients, and characteristics of revascularization procedures [17-18]. However, in our study, the low rates of revascularization were found to be due to patients’ refusal of surgery, low socioeconomic status, and high-risk patient profiles.

Limitations

This study has a few limitations. First, we were unable to classify patients based on symptomatology since symptoms could influence surgery preference. Secondly, we could not specify individual risk factors for surgery that led to a surgeon’s refusal. Thirdly, we did not take syntax scoring into account. The fourth limitation is that our study was limited to a single center. Our fifth limitation is that we did not follow up on our patients beyond six months; hence, these patients’ final fate remains unknown. We recommend that future studies take note of these limitations and try to address them so that the shortcomings in our study could be remedied.

## Conclusions

This study demonstrated that a limited number of patients underwent CABG, even though they had clear indications for the procedure. The patient’s high-risk profile, patient unwillingness, and high cost of the procedure were the primary reasons behind this downward trend. Our study results may have significant medical and social implications and can stimulate discussions about decision-making approaches among healthcare professionals and patients with severe CAD. Although many individuals may prefer PCI because it is much less invasive, cardiologists and cardiothoracic surgeons must get involved in the decision-making process to determine the most appropriate procedure for patients. Patients should be educated on all possible treatment options. Larger studies are required so that the determinants behind the decline in CABG surgeries could be inspected on a larger scale and dealt with in the best way possible.
